# Processing of (Co)Poly(2-oxazoline)s by Electrospinning and Extrusion from Melt and the Postprocessing Properties of the (Co)Polymers

**DOI:** 10.3390/polym12020295

**Published:** 2020-02-02

**Authors:** Wojciech Wałach, Natalia Oleszko-Torbus, Alicja Utrata-Wesołek, Marcelina Bochenek, Ewa Kijeńska-Gawrońska, Żaneta Górecka, Wojciech Święszkowski, Andrzej Dworak

**Affiliations:** 1 Centre of Polymer and Carbon Materials, Polish Academy of Sciences, 34 M. Curie-Skłodowskiej St., 41-819 Zabrze, Poland; noleszko@cmpw-pan.edu.pl (N.O.-T.); autrata@cmpw-pan.edu.pl (A.U.-W.); mbochenek@cmpw-pan.edu.pl (M.B.); adworak@cmpw-pan.edu.pl (A.D.); 2 Faculty of Materials Science and Engineering, Warsaw University of Technology, 141 Woloska St., 02-507 Warsaw, Poland; ewa.kijenska@pw.edu.pl (E.K.-G.); gorecka.zaneta@gmail.com (Ż.G.); wojciech.swieszkowski@inmat.pw.edu.pl (W.Ś.)

**Keywords:** poly(2-isopropyl-2-oxazoline), poly(2-oxazoline)s, electrospinning, melt extrusion, nanofibres, polymer processing

## Abstract

Poly(2-oxazoline) (POx) matrices in the form of non-woven fibrous mats and three-dimensional moulds were obtained by electrospinning and fused deposition modelling (FDM), respectively. To obtain these materials, poly(2-isopropyl-2-oxazoline) (PiPrOx) and gradient copolymers of 2-isopropyl- with 2-n-propyl-2-oxazoline (P(iPrOx-nPrOx)), with relatively low molar masses and low dispersity values, were processed. The conditions for the electrospinning of POx were optimised for both water and the organic solvent. Also, the FDM conditions for the fabrication of POx multi-layer moulds of cylindrical or cubical shape were optimised. The properties of the POx after electrospinning and extrusion from melt were determined. The molar mass of all (co)poly(2-oxazoline)s did not change after electrospinning. Also, FDM did not influence the molar masses of the (co)polymers; however, the long processing of the material caused degradation and an increase in molar mass dispersity. The thermal properties changed significantly after processing of POx what was monitored by increase in enthalpy of exo- and endothermic peaks in differential scanning calorimetry (DSC) curve. The influence of the processing conditions on the structure and properties of the final material were evaluated having in a mind their potential application as scaffolds.

## 1. Introduction

A great number of studies have focused lately on the processing of polymers into two- and three-dimensional matrices with strictly defined size and shape. Such matrices are of great interest in materials science because of their multiple applications in different fields, like electronic [1], or controlled drug delivery [2]. Especially, a great interest in recent years is the processing of polymers into materials that can be applied as scaffolds in tissue regeneration [3,4,5,6,7,8]. Among synthetic and natural polymers, mainly polycaprolactone (PCL) [9], poly(l-lactic acid) (PLA) [10], poly(lactic-co-glycolic) acid (PLGA) [11], silk [12], collagen [13], hyaluronic acid [14] or chitosan [15], were utilised for the fabrication of matrices applied as scaffolds for tissue regeneration.

A simple and convenient technique that provides matrices in the form of two-dimensional non-woven fibrous mats is electrospinning of polymer solutions. The non-woven fibrous mat is composed of fibres with very small diameters, typically in the range of few a nanometres to micrometres, thus it possess large surface-area-to-volume ratios and high porosity. The morphology of the fibres, such as the diameter or uniformity, can be tailored by selecting the processing parameters, e.g., polymer concentration, type of solvent (surface tension and electrical conductivity), applied voltage and flow rate, or ambient conditions, such as humidity [16]. The possibility of scaling up the production makes this technique attractive for various applications. In addition to biomedicine [17,18], the best known and described areas where electrospun polymer fibres can be applied are filtration, electronics, membranes, optics and sensors [19,20].

Matrices with strictly defined parameters can also be obtained using a variety of innovative 3D printing technologies [21,22,23]. One well-known method is the extrusion of polymers from melt. By using this method, two- and three-dimensional moulds can be obtained. Basically, the polymer is heated to its melting point and then it is extruded through the nozzle to form a fibre, which is placed gradually, layer by layer, to create an object in accordance with the previously designed model. It is a solvent-free process that results in moulds of strictly defined sizes, shapes and porosities. A limitation of this technique is related to the stability of the polymer at the selected processing temperature.

In recent years, poly(2-oxazoline)s (POx), analogues of polypeptides, have become an essential class of polymers due to their prospective applications in various fields, e.g., medicine and biotechnology. They are nontoxic, exhibit good biocompatibility, do not accumulate in tissues and are rapidly cleared from the blood [24,25,26,27,28]. POx are obtained via cationic ring-opening polymerisation, where their architectures, chemical structures and compositions may be easily controlled [29,30]. Consequently, polymers with properties that can be adjusted in a precise manner can be obtained. The nature of the side chains in the POx determines the behaviour of the polymers in solution, but it also determines their thermal properties and their ability to crystallise in the condensed state. Poly(2-oxazoline)s with three or more carbon atoms in the side chain are known to crystallise in bulk and/or in aqueous or water/organic solvent solutions [31,32,33]. Among this class of polymers, poly(2-isopropyl-2-oxazoline) (PiPrOx) is extensively studied due to its high ability to crystallise.

Although a great number of studies have focused on the synthesis of POx with different architectures, properties and potential applications (e.g., as drug carriers, supports facilitating the culture of cells or antifouling surfaces [34,35,36,37]), there is not extensive research on the processing of these polymers into 2D or 3D materials with strictly defined sizes and shapes that could be potentially applied as scaffolds.

The literature data on the formation of non-woven fibrous mats based on POx is limited, and it mainly concerns the electrospinning of commercially available poly(2-ethyl-2-oxazoline) (PEtOx) of high molar mass and broad dispersity (Aquazol®) [38,39,40,41,42,43]. The first study on the optimisation of electrospinning conditions of an aqueous solution of PEtOx (*M*_w_ of ~500,000 g/mol) was described by Buruaga et al. [38]. In this article, the first, but not successful, attempts of receiving non-woven mats from PEtOx dissolved in organic solvents (DMF and THF) are described. In turn, George et al. [39] obtained ultrafine fibres of cobalt oxide (Co_3_O_4_) by combining the electrospinning method with high-temperature calcination from the precursor of PEtOx (*M*_w_ of ~500,000 g/mol)/cobalt acetate tetrahydrate in water. High molar mass PEtOx was also used as a potential carrier for the electrospinning of enzymes [40]. Optimisation of the process demonstrated that enzymes were more active after spinning with polymer than spinning alone. Hochleiter et al. [41] used a method referred to as “melt electrospinning writing” to obtain high-definition fibrous scaffolds from PEtOx of lower molar mass (*M*_w_ of ~50,000 g/mol) but high dispersity (*Ð* of ~3–4). They performed systematic studies on the influence of the processing parameters on fibre formation, and the resultant fibres were found to have large diameters of 8 to 130 µm. The only studies of the electrospinning of an aqueous solution of PEtOx with low molar mass and low dispersity were described by Kalaoglu-Altan et al. [42] and Stubbe et al. [43]. In the first case, a mixture of 2-ethyl-2-oxazoline-based copolymers with alkene pendant groups (*M*_n_ of 18,000 g/mol; *Ð* = 1.14), a thiol-containing crosslinker, and a photoinitiator were subjected to in situ UV-cross-linking after electrospinning [42]. In the latter case, the electrospinning parameters for PEtOx with a *M*_n_ of ~40,000 and 70,000 g/mol (estimated using poly(methyl methacrylate) calibration) and *Ð =* 1.15 and 1.16, respectively, were compared to the conditions needed to obtain fibres from Aquazol® [43]. It was shown that solutions of POx with low molar mass required higher concentrations for electrospinning as a result of the lower solution viscosities. However, interestingly, the parameters for obtaining fibres from PEtOx with *M*_n_ = 70,000 g/mol were only slightly different from the optimal parameters determined for Aquazol®. Too high viscosity, as a result of too high of a molar mass (*M*_w_ of 500,000 g/mol), is not been desired, and has led to beaded fibres or clogging of the spinneret at the tip.

Considering the formation of POx three-dimensional matrices by extrusion from melt, only PEtOx with a wide range of molar masses (50,000, 200,000 and 500,000 g/mol) was processed by this technique [44]. The polymer mixed both with highly water-soluble and poorly water-soluble drugs (metoprolol tartrate and fenofibrate) was processed by hot melt extrusion followed by injection moulding to obtain tablets for oral drug formulations. A process temperature of 160 °C was applied, which was below the decomposition temperature of PEtOx, and it allowed to obtain transparent tablets with smooth surfaces. Also, a small amount (1 wt %) of amphiphilic block copolymer based on 2-nonyl- and 2-methyl-2-oxazoline mixed with poly(l-lactide-co-glycolide) (molar ratio of 85:15) was used to obtain scaffolds via precision extruding deposition [45].

During the production of two- or three-dimensional matrices, different factors that may have a potential impact on the processability and stability of the formed materials must be considered. For example, in the electrospinning technique, an effect of electrospray instead of fibre formation will be achieved, when too low concentration of the polymer in the solution, an inappropriate voltage or flow rate is chosen [46]. In the case of extrusion from melt, due to the pressure used to push the polymer through the nozzle and the shear forces induced by the rotation of the screw, the temperature of processing may increase drastically. This may possibly lead to degradation of the polymer; thus, the temperature must be accurately monitored. To omit this problem, plasticising of the polymers, leading to a decrease in the polymer flow temperature, can be performed.

Changes in the physicochemical properties of the polymers during processing (such as decomposition of the polymer or an increase in the crystalline fraction) are difficult to predict. Having in a mind the stability of the final product, properties of the polymer after processing should also be investigated.

In this work, we obtained non-woven fibrous mats and three-dimensional moulds by the processing of poly(2-isopropyl-2-oxazoline) and gradient copolymers of 2-isopropyl- with 2-n-propyl-2-oxazoline. For this purpose, electrospinning and extrusion from melt were applied. For the first time, the poly(2-oxazoline) other than PEtOx was applied in electrospinning process. In these studies, POx of low molar masses and low dispersity values were used. The electrospinning conditions were optimised in both water and hexafluoro-2-propanol (HFP). Also, the extrusion from melt of the POx was briefly optimised. The properties of the POx after electrospinning and extrusion from melt were determined to evaluate the influence of the processing conditions on the structure and properties of the final material.

## 2. Materials and Methods

Materials: Isobutyronitrile (99.6%, Sigma-Aldrich, Steinheim, Germany), n-butyronitrile (>99%, Fluka, Steinheim, Germany), 2-aminoethanol (99%, Aldrich), cadmium acetate (>98%, Fluka), methyl 4-nitrobenzenesulfonate (99%, Aldrich) and propylamine (>99%, Aldrich) were used as received. Water and hexafluoro-2-propanol (HFP) were filtrated prior to use. Acetonitrile (ACN, 99.9%, POCH, Gliwice, Poland) was dried over CaH_2_ and distilled under dry argon. The protocol for the synthesis of 2-isopropyl-2-oxazoline (iPrOx) and 2-n-propyl-2-oxazoline (nPrOx) was the same as that described previously [47]. Briefly, isobutyronitrile or n-butyronitrile was mixed in an equimolar amount with 2-aminoethanol and heated under reflux in the presence of cadmium acetate. The resulting monomers were dried over KOH, distilled under reduced pressure, dried over CaH_2_, and distilled again. For the polymerisations, a fraction of monomers with purity higher than 99.8% (GC) was applied.

Synthesis of iPrOx homopolymers and iPrOx/nPrOx copolymers: Cationic ring-opening polymerisation was used for the synthesis of the POx. The applied procedure was the same as that described in [48]. Briefly, initiator (methyl 4-nitrobenzenesulfonate) was introduced in argon atmosphere into previously dried ampoule equipped with the glass-teflon valve and dissolved in dried acetonitrile. The freshly distilled monomer (iPrOx) or mixture of comonomers (iPrOx/nPrOx) was introduce directly to the solution of the initiator. Theoretical degrees of polymerisation of 170 and 350 were assumed for PiPrOx, while 90 and 450 were assumed for the copolymers of iPrOx and nPrOx. The polymerisations were carried out in acetonitrile at 75 °C to conversion of monomers higher than 98% (checked by gas chromatography). The living polymer chains were terminated by the addition of n-propylamine. After evaporation of the acetonitrile, the polymers were dissolved in deionised water and the solution was filtered and dried by lyophilisation.

Electrospinning: The basic set-up for electrospinning was composed of a syringe pump (KD Scientific, Holliston, MA, USA), a high voltage power supply (Gamma High Voltage Research, Ormond Beach, FL, USA) and a flat steel plate collector covered with aluminium foil. During electrospinning of aqueous PiPrOx solutions, a voltage of 16–20 kV was applied, whereas for PiPrOx dissolved in HFP, 10–14 kV was used. For each concentration, the following four different flow rates were applied; 0.5, 0.8, 1 and 1.5 mL/h. The needle-to-collector distance was set at 12 cm for all experiments. All solutions were ejected through 27G blunted steel needles. Electrospun samples collected on aluminium foil were kept in a vacuum drier (50 mbar, 25 °C) prior to examination. Electrospinning set is shown in Figure S1 in a Supplementary Materials. 

Extrusion from melt: To obtain three-dimensional moulds, Bioscaffolder (SYSENG, Salzgitter-Bad, Germany) was used. The formation of moulds was performed at 155–160 °C for iPrOx/nPrOx copolymers and at 205 °C for iPrOx homopolymers. A pressure of 5 bar and a rotation of the Auger screw of 160 rpm was applied during the extrusion of (co)polymers. Nozzles with the inner diameter of 250 and 410 μm for iPrOx/nPrOx copolymers and 330 μm for iPrOx homopolymer were used. The XY printing speed was 30 mm/min. The Bioscaffolder system used for the extrusion from melt of (co)polymers is shown in Figure S2 in a Supplementary Materials. 

Characterisation of the obtained polymers before and after processing: The conversion of monomers was determined on the Varian 3400 gas chromatograph equipped with the FID detector, injector with the sample splitting system (split ratio 98:2) and J&W Scientific DB5 column (30 m × 0.32 mm; film thickness 0.25 µm). Samples of 0.25 mL were diluted with the 2 mL of acetonitrile with the internal standard (p-toluene 2% V/V) and 1 µL of the solution was injected. The conversion of the monomer was calculated with respect to the samples taken directly before the polymerisation.

The molar masses and dispersities (*Ð*) of the (co)polymers were determined using a gel permeation chromatography system with a multiangle laser light scattering detector (GPC MALLS, Santa Barbara, CA, USA) (DAWN EOS, Wyatt Technologies, λ = 658 nm) and a refractive index detector (Δn-1000 RI WGE DR Bures, λ = 620 nm). Measurements were carried out in DMF (with 5 mmol/L of LiBr; flow rate of 1 mL/min) using 10 µm PSS 100 Å, 1000 Å and 3000 Å GRAM columns. The refractive index increment (*dn/dc*) was independently measured in DMF. 

Turbidimetric measurements of the aqueous solutions of (co)polymers (5 g/L) were monitored at λ = 500 nm as a function of temperature using a Specord 200plus UV–Vis spectrophotometer (Analytik Jena, Jena, Germany) equipped with a programmable thermo-controller. Transmittance values were recorded every 1 °C after 60 s of temperature stabilisation. The T_CP_ value was defined as the temperature at which the transmittance of the polymer solution reached 50% of its initial value.

Differential scanning calorimetry (DSC) measurements were performed using a TA-DSC Q2000 apparatus (TA Instruments, Newcastle, DE, USA) in the range of −50 to 250 °C under a nitrogen atmosphere (flow rate of 50 mL/min). The heating rate for the samples was 10 °C/min. The instrument was calibrated with high purity indium. The data were collected and then analysed using Universal Analysis 2000, Universal V4.5a software.

SEM studies were performed using a Quanta 250 FEG and PhenomProX (FEI Company, Hillsboro, OR, USA) high resolution environmental scanning electron microscope operated at an acceleration voltage of 10 kV in backscattered electron mode. Prior to the observations, all samples were sputter-coated with a 7 nm layer of gold.

The mechanical properties of the non-woven mats were analysed with an atomic force microscope (AFM) (Dimension Icon model) by Bruker (Santa Barbara, CA, USA). Young’s modulus was determined in quantitative nanomechanical mapping mode at four measuring points along the fibril. Measurements were made using a non-calibrated needle; thus, the obtained modules are given as relative values.

## 3. Results and Discussion

PiPrOx is known from its high ability to crystallise in both the condensed state and in solution [49]. Although its cloud point temperature (T_CP_) in aqueous solution is close to that of the human body, making it attractive for many applications, the presence of a crystalline phase is often not desirable in some systems. It was shown [48] that copolymerisation of iPrOx with n-propyl-2-oxazoline in an amount of 50 mol% significantly decreased the ability of the copolymers to crystallise, compared to the homopolymers of iPrOx. The aim of these studies was to determine whether iPrOx-based (co)polymers could be processed by electrospinning and extrusion from the melt techniques and to determine how processing affects their properties.

### 3.1. Synthesis and Characterisation of (Co)poly(2-oxazoline)s

Poly(2-isopropyl-2-oxazoline)s and copolymers of 2-isopropyl-2-oxazoline with 2-n-propyl-2-oxazoline were obtained via cationic ring-opening polymerisation initiated by methyl 4-nitrobenzenesulfonate. Copolymerisation of iPrOx with nPrOx led to copolymers of gradient distribution of comonomers. The precise kinetics of this reaction have been shown elsewhere [50]. The symbols and characterisation data of the obtained (co)polymers are summarised in Table 1. ^1^H NMR spectra are presented in Figure S3 in a Supplementary Materials. 

**Figure 1:**
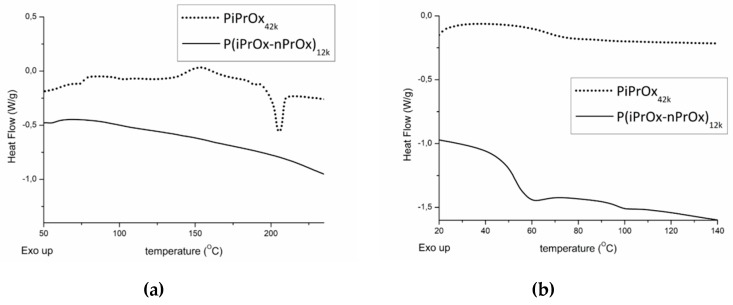
Differential scanning calorimetry (DSC) traces of PiPrOx_42k_ and P(iPrOx–nPrOx)_12k_. (**a**) First run and (**b**) second run after quenching from melt (220 °C) in liquid nitrogen. Heating rate of 10 °C/min.

Chromatograms for all (co)polymers were monomodal and the dispersity Ð was low, except for the copolymer with a molar mass of more than 50,000 g/mol. The copolymer with a dispersity of 1.32 was prepared with a high monomer concentration, which resulted in a high viscosity at the end of the polymerisation and, thus, higher dispersity. A good agreement between the molar masses and theoretical values was achieved, indicating the proper control of the polymerisation process. Based on the turbidimetric measurements, all iPrOx-based (co)polymers dissolved in water exhibited thermosensitivity, and the phase transition was completely reversible (data not shown). The thermal properties of the (co)polymers were studied by DSC (Figure 1).

For PiPrOx_21k_ (DSC curve is shown in Figure S4 in Supplementary Materials) and PiPrOx_42k_ during the first DSC run, an exothermic peak could be detected in the range of 130 to 180 °C, with the maximum located at approximately 150 °C. The heat of the observed effect was attributed to the crystallisation of PiPrOx during the measurement, and it was approximately of 15 J/g. At a temperature of ~200 °C, the visible endothermic peak occurred due to the melting of the freshly crystallised sample. The enthalpy of melting was the same as the heat of crystallisation. For P(iPrOx–nPrOx)_12k_, neither exothermic nor endothermic peaks could be seen in the DSC trace, indicating significantly less ability to crystallise compared to PiPrOx. Afterwards, the samples were subjected to quenching from melt (220 °C) in liquid nitrogen and a second DSC run was performed. The glass transition of PiPrOx could be seen at a temperature of approximately 70 °C. Copolymerisation of iPrOx with nPrOx led to a considerable plasticising effect, and a decrease in T_g_ to ~50 °C was observed. These values were consistent with data from the literature [48].

The obtained (co)poly(2-oxazoline)s were subjected to processing by electrospinning and extrusion from melt to obtain both flat, two-dimensional non-woven mats and three-dimensional moulds.

### 3.2. Electrospinning of (Co)poly(2-oxazoline)s

The obtained (co)poly(2-oxazoline)s of relatively low molar mass (from 12,600 to 51,000 g/mol) and low dispersity were subjected to the electrospinning experiments. Optimisation of the electrospinning conditions was carried out using poly(2-isopropyl-2-oxazoline). A series of solutions of PiPrOx_21k_ and PiPrOx_42k_ in water and HFP (commonly used solvent in the electrospinning of polymers) with different concentrations were prepared. Non-woven mats of various fibre morphologies, diameters and uniformities were obtained and analysed.

#### 3.2.1. Fibre Morphology

**Figure 2:**
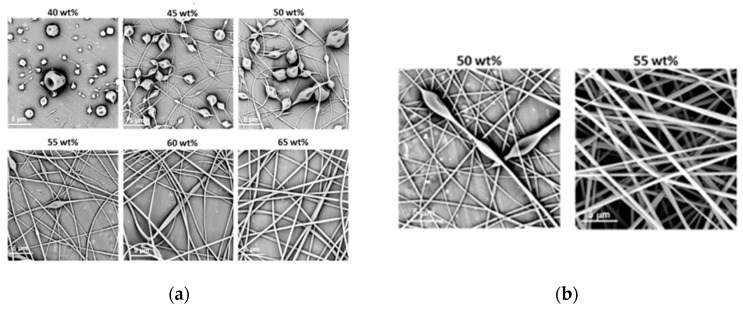
Morphology of structures after electrospinning of (**a**) PiPrOx_21k_ and (**b**) PiPrOx_42k_ at different concentrations (wt %: weight percent) in water (flow rate of 1 mL/h).

The morphologies of the structures obtained during electrospinning of aqueous solutions of PiPrOx_21k_ and PiPrOx_42k_ with increasing concentration are presented in Figure 2.

It was observed that in the case of the aqueous solution of PiPrOx_21k_ and PiPrOx_42k_, at concentrations below 40 wt % and 35 wt %, respectively, a so-called electrospray effect was observed. Electrospinning of aqueous solutions of PiPrOx_21k_ at concentrations from 45 to 50 wt % resulted in the formation of beads or beaded fibres. The same morphology was observed for poly(2-isopropyl-2-oxazoline) of higher molar mass (PiPrOx_42k_); however, in this case, the concentration was 45 wt %. Upon increasing the concentration of the aqueous solution up to 60 wt % for PiPrOx_21k_ and up to 50 wt % for PiPrOx_42k_, the shape of the fibres changed into a spindle-like structure. However, regular beads could still be detected in some parts of the fibres. Homogeneous fibres were observed for concentrations above 60 wt % for the aqueous solution of PiPrOx_21k_ and above 50 wt % in case of PiPrOx_42k_. The highest concentration at which it was still possible to obtain regular, well-defined fibres was 70 wt % for PiPrOx_21k_ and 60 wt % for PiPrOx_42k_. A similar concentration range was applied during electrospinning of poly(2-ethyl-2-oxazoline) of a comparable molar mass [43]. A detailed comparison revealed that the well-defined fibres were formed at concentrations of 55–60 wt % for aqueous solutions of PEtOx with *M*_n_ = 37,500 g/mol and PiPrOx_42k_ with *M*_n_ = 42,000 g/mol. Based on the results obtained in this work, it should be noted that PiPrOx of low molar mass and narrow dispersity can form non-woven fibres when electrospun from an aqueous solution.

**Figure 3:**
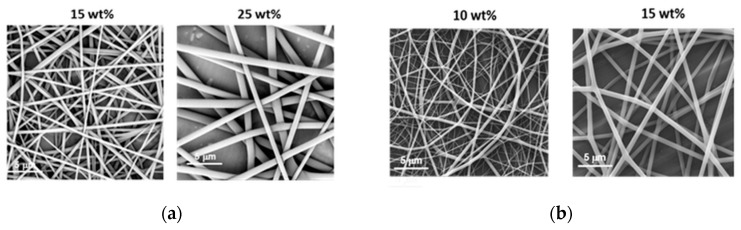
Morphology of (**a**) PiPrOx_21k_ and (**b**) PiPrOx_42k_ fibres obtained at different concentrations in HFP (flow rate of 1 mL/h).

Much lower concentrations could be applied to obtain fibrils from PiPrOx_21k_ and PiPrOx_42k_ in the organic solvent HFP (Figure 3).

Based on the obtained results, note that in the case of PiPrOx_21k_ in HFP, the effect of electrospraying was observed only for the lowest applied concentration of 10 wt %, whereas regular fibres were formed when solutions at concentrations of at least 15 wt % were applied. For PiPrOx_42k_ (of the higher molar mass) in HFP, the concentration necessary for fibre formation has lowered to 10 wt %. The concentration range of PiPrOx_21k_, enabling homogeneous fibre formation, was broader (15–35 wt %) than that of PiPrOx_42k_ (10–20 wt %).

It is known that among the many parameters that influence the electrospinning process for producing non-woven mats, the surface tension and conductivity of the solvent are crucial. To force the portion of the polymer solution to leave the spinneret at the tip of the electrospinning machine, the charges accumulated on the surface of the drop of solution must overcome the forces of its surface tension. The surface tension of HFP (~16 mN/m at 20 °C) is much lower than that of water (~72 mN/m at 20 °C); therefore, the charges accumulated on the droplet surface are more likely to "overwhelm" the force of its surface tension, and the formation of fibres occurs at lower concentrations in HFP than in water. The only studies on the electrospinning of POx in organic solvents were carried out by Buruaga [38] and Kalaoglu-Altan [42]. The electrospinning of PEtOx dissolved in DMF resulted in thin fibres of beaded morphology, which was attributed to the limited solubility of the polymer in DMF and the high value of its surface tension (~37 mN/m). On the other hand, electrospinning of PEtOx in THF was difficult, despite its lower value of surface tension (~26 mN/m), due to its fast evaporation in the hanging droplets. This induced high viscosity of the solution and clogging of the spinneret at the tip. However, the EtOx-based copolymer dissolved in the mixture of DMF and THF (1:1), with in situ UV-cross-linking during electrospinning, was found to form bead-free fibrils with an average diameter of ~540 nm (+/−230 nm). To the best of our knowledge, the results presented in our work are the first where application of an appropriate organic solvent in the electrospinning of poly(2-oxazoline) led to stable non-woven homogeneous fibrils, without their stabilisation by crosslinking.

#### 3.2.2. Fibre Diameter and the Uniformity of Fibre Diameter

**Figure 4:**
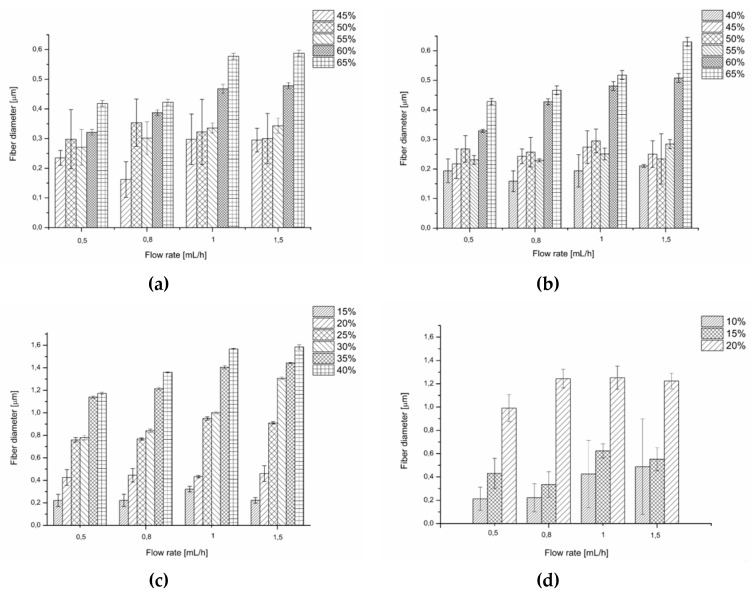
Comparison of the diameter and the uniformity of the diameter for fibres of (**a**) PiPrOx_21k_ in water, (**b**) PiPrOx_42k_ in water, (**c**) PiPrOx_21k_ in HFP, and (**d**) PiPrOx_42k_ in HFP.

Generally, for the solvents applied in this work, within given flow rate the diameters of the fibres increased with an increasing concentration of the PiPrOx solution, which is consistent with literature data for various electrospun polymers (Figure 4).

Generally, for both water and HFP solutions, within the given concentration, an increase in the flow rate caused an increase in the fibre diameter. For a given flow rate, the fibres of the highest diameter (up to 1.6 μm) were obtained when PiPrOx was dissolved in HFP, although at lower concentrations than in water. This was due to the favourable combination of the surface tension and conductivity values for this solvent, compared to water, which is described above.

In most cases, for aqueous solutions the fibre diameter distribution was high for applied concentrations in range of 40 to 55 wt %, regardless of the molar mass of PiPrOx and the applied flow rate. Uniformity of the diameter of the fibres was observed in aqueous solution with a concentration between 60 and 65 wt %. Conversely, fibres obtained from solutions in HFP were more homogeneous in diameter, starting from a concentration of 25 wt % for PiPrOx_21k_ and 15 wt % for PiPrOx_42k_.

**Figure 5:**
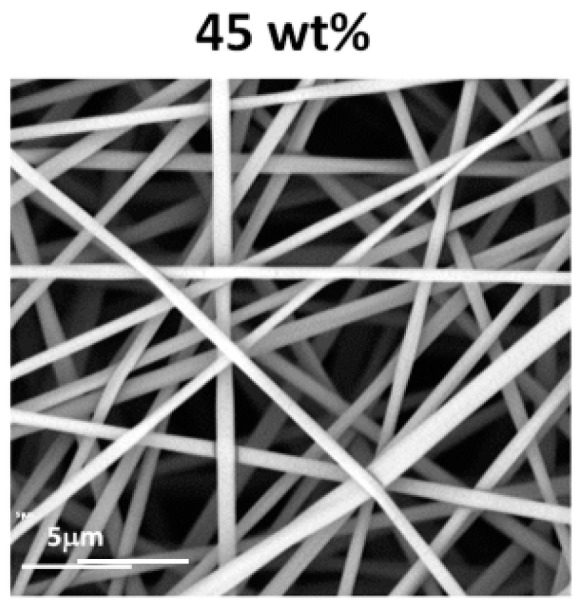
Morphology of P(iPrOx–nPrOx)_51k_ fibres obtained at a concentration of 45 wt % in water (flow rate of 1 mL/h).

Based on the optimisation of the electrospinning conditions for PiPrOx, processing of the exemplary P(iPrOx–nPrOx)_51k_ copolymer was also carried out by applying a concentration of the copolymer in water of 45 wt % and a flow rate of 1 mL/h. Non-woven mats of regular fibre morphology were obtained (Figure 5).

#### 3.2.3. Elasticity of Non-woven Mats

An adequate elasticity of electrospun mats is generally a prerequisite for their practical usage. To apply non-woven mats as matrices in biomedical engineering, they need to mimic the flexibility of the extracellular matrix to allow the cells to easily grow and proliferate. The governing factors that affect the elasticity of non-woven mats, beyond the porosity, average fibre diameter or fibre orientation, are the thermal and crystalline properties of the polymer used for electrospinning.

**Figure 6:**
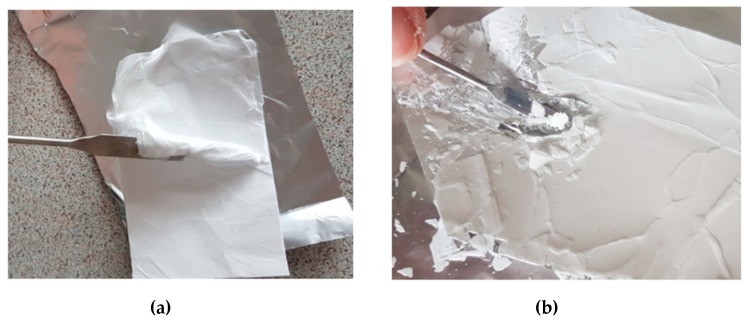
(**a**) P(iPrOx–nPrOx)_51k_ and (**b**) PiPrOx_42k_ non-woven mats.

Non-woven mats of similar fibre diameter and morphology, obtained from PiPrOx_42k_ in HFP at a concentration of 15 wt % and P(iPrOx–nPrOx)_51k_ in water at a concentration of 45 wt %, differed significantly in terms of elasticity. P(iPrOx–nPrOx)_51k_ non-woven mats were more elastic compared to those obtained from PiPrOx_42k_. They could be rolled up and easily removed from aluminium foil, on which they were prepared (Figure 6a). Conversely, PiPrOx non-woven mats were stiff and fragile, and removing them from aluminium foil caused damage to the uniformity of the mat (Figure 6b).

Due to the high fragility of PiPrOx non-woven mats, it was impossible to determine their mechanical properties by dynamic mechanical analysis. However, with the quantitative nanomechanical mapping performed by AFM, the relative Young’s modulus could be measured (Figure S5 in a Supplementary Materials). These relative values were approximately of 100 kPa for P(iPrOx–nPrOx)_51k_ and 320 kPa for PiPrOx_42k_ (average from four measuring points). More than three times higher values of the relative Young’s modulus for PiPrOx fibres than for P(iPrOx–nPrOx) fibres confirms the greater stiffness and brittleness of these non-woven mats. Differences in elasticity and stiffness of obtained non-woven mats are probably related to the amount of crystalline phase in the electrospun polymers. As discussed above and confirmed by the DSC traces (Figure 1), PiPrOx is much more prone to crystallisation than the copolymers of iPrOx with nPrOx; therefore, mats of PiPrOx_42k_ were more stiff and fragile. 

### 3.3. Extrusion From the Melt of (Co)Poly(2-oxazoline)s

**Figure 7:**
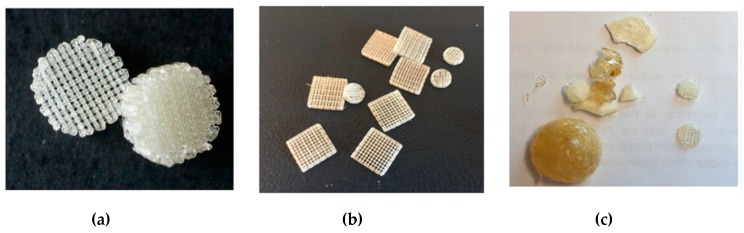
Three-dimensional moulds obtained by fused deposition modelling (FDM) of (**a**) P(iPrOx–nPrOx)_12k_, (**b**) P(iPrOx–nPrOx)_51k_ and (**c**) PiPrOx_21k_ together with a fraction of the polymer that blocked the system.

Fused deposition modelling (FDM) is one of the rapid prototyping technique that involves the formation of a three-dimensional object with the molten polymer [51,52,53]. Basically, the polymer is heated to its melting point and then extruded through the nozzle to form a fibre that is placed gradually, layer by layer, to form an object in accordance with the previously designed model. In these studies, multi-layer moulds of cylindrical shape with a diameter of 7 and 10 mm and of cubical shape with a side length of 10 mm were obtained. The distance between fibres among single layer was 500 and 820 μm, whereas the angle between fibres in subsequent layers was 90°. The formation of moulds was performed at 160 °C for P(iPrOx–nPrOx)_12k_ and P(iPrOx–nPrOx)_51k_ and at 205 °C for PiPrOx homopolymers. When applying a pressure of 5 bar during extrusion through the nozzle and a rotation of the screw of 160 rpm, both P(iPrOx–nPrOx)_12k_ and P(iPrOx–nPrOx)_51k_ could be easily extruded, and moulds were obtained (Figure 7a,b). After the formation of dozens of moulds, the fraction of the copolymers that remained in the nozzle became yellowish and blocked the system, thus preventing the subsequent formation of moulds. Most likely, the copolymers that were heated at an extrusion temperature of 160 °C for a prolonged time period (several hours) had degraded.

When applying the same pressure and rotation of the screw during the extrusion of PiPrOx, the efficiency of the process was much lower, and only several moulds could be obtained (Figure 7c). The polymer remained in the extrusion system almost immediately blocked the nozzle and became yellowish. This fraction was formed probably due to thermal degradation of PiPrOx and further extruding was impossible.

It seemed that this technique was not fully appropriate for the formation of moulds from poly(2-isopropyl-2-oxazoline). Moulds obtained from iPrOx/nPrOx copolymers were stable, elastic and non-fragile.

### 3.4. Characterisation of (Co)poly(2-oxazoline)s after Processing

#### 3.4.1. Molar Mass Evaluation

**Figure 8:**
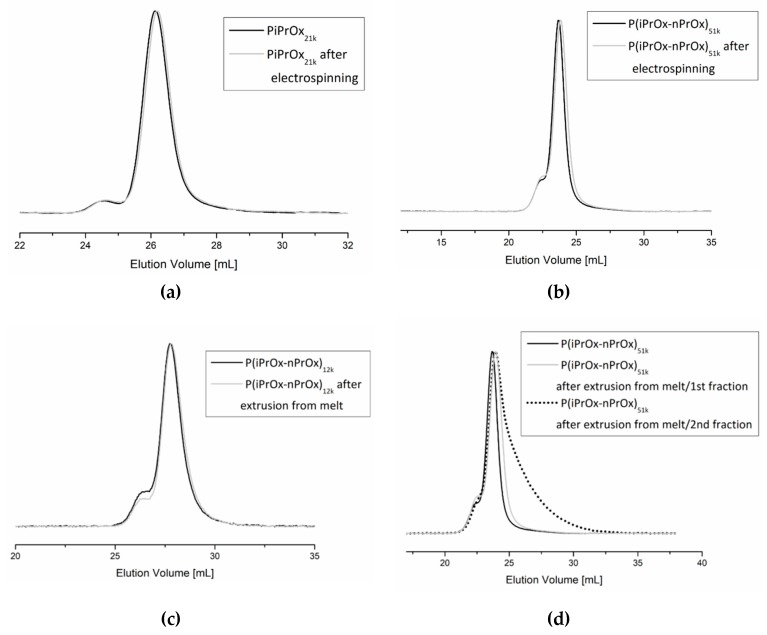
SEC traces of (**a**) PiPrOx_21k_ and (**b**) P(iPrOx–nPrOx)_51k_ before and after electrospinning and of (**c**) P(iPrOx–nPrOx)_12k_ and (**d**) P(iPrOx–nPrOx)_51k_ before and after extrusion from melt.

Fibrous mats obtained by electrospinning and three-dimensional moulds obtained by FDM were investigated in terms of the possible degradation of the polymer during processing. The molar mass and molar mass distribution of the polymers after processing were analysed by size exclusion chromatography. Exemplary non-woven mats obtained from electrospinning of PiPrOx_21k_ in HFP at a concentration of 25 wt % (flow rate of 1 mL/h) and of P(iPrOx–nPrOx)_51k_ in water at a concentration 45 wt % (flow rate of 1 mL/h) were examined (Figure 8a,b). As the extrusion from melt was found to be non-efficient for PiPrOx, only moulds obtained from P(iPrOx–nPrOx)_12k_ and P(iPrOx–nPrOx)_51k_ were analysed by GPC (Figure 8c,d).

The chromatograms of PiPrOx_21k_ and P(iPrOx–nPrOx)_51k_ after electrospinning from water or HFP were monomodal, with the small bend from the higher molar mass exactly the same as in polymers before electrospinning. The *M*_n_ for these polymers were 21,100 g/mol (*Ð* = 1.04) and 47,000 g/mol (*Ð*=1.30), respectively, which is nearly the same as the starting molar masses. This means that during the process of electrospinning there was no significant degradation of the polymer chain.

Similarly, no degradation of the polymer was observed for P(iPrOx–nPrOx)_12k_ and P(iPrOx–nPrOx)_51k_ copolymers after extrusion from melt. The molar masses of these copolymers after extrusion were 11,500 and 48,000 g/mol, respectively. However, as mentioned earlier, when copolymers were heated at the temperature of extrusion (160 °C) for a prolonged time, the fraction of the polymer that remained in the nozzle became yellowish and the polymer degraded. A molar mass of 20,000 g/mol and a wide molar mass dispersity *Ð* of 1.7 were observed (designated in Figure 8d as P(iPrOx–nPrOx)_51k_ after extrusion from the melt/2^nd^ fraction).

#### 3.4.2. Evaluation of Thermal and Crystalline Properties

**Figure 9:**
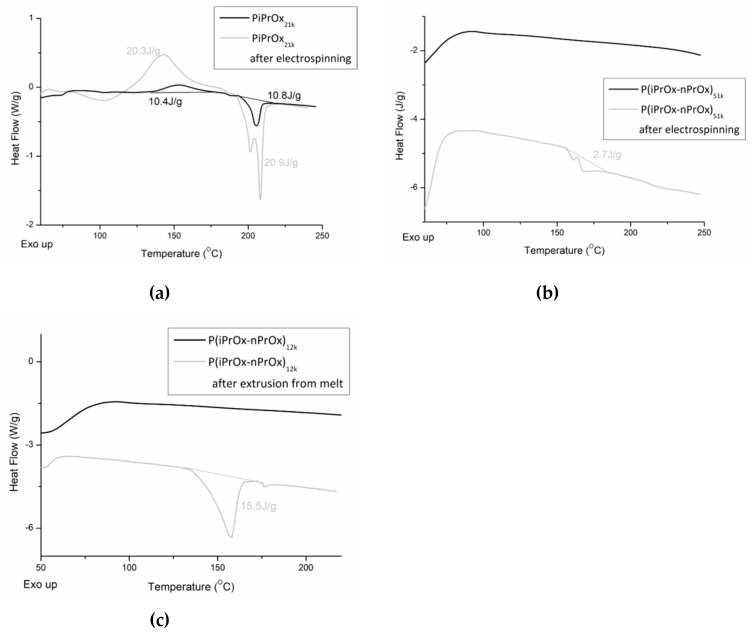
DSC traces of (**a**) PiPrOx_21k_ and (**b**) P(iPrOx–nPrOx)_51k_ before and after electrospinning and DSC traces of (**c**) P(iPrOx–nPrOx)_12k_ before and after extrusion from melt.

The changes in the thermal and crystalline properties of the fibrous mats and the three-dimensional moulds after processing were evaluated using DSC. Non-woven mats obtained from the electrospinning of PiPrOx_21k_ in HFP at a concentration of 25 wt % (flow rate of 1 mL/h) (Figure 9a) and of P(iPrOx–nPrOx)_51k_ in water at a concentration of 45 wt % (flow rate of 1 mL/h) (Figure 9b) were applied for this evaluation. Also, moulds of P(iPrOx–nPrOx)_12k_ were analysed (Figure 9c).

After electrospinning, the thermal properties of all (co)poly(2-oxazoline)s changed significantly. In the case of PiPrOx, both the range of crystallisation temperature and melting in the first run and the glass transition in the second run were the same as before electrospinning. However, the enthalpy of crystallisation and melting increased from ~10 J/g to ~20 J/g. Also, it could be seen that the melting peak was not symmetric, as in the case of PiPrOx before electrospinning. Such results may be attributed to the orientation of the macromolecular chains within the polymer in the longitudinal fibre direction under an electrostatic field during the electrospinning process, which may promote crystallisation. Such effect was also observed for other polymers [54,55,56,57]. Similarly, P(iPrOx–nPrOx) copolymers were amorphous before electrospinning, but a nonsymmetric peak (ΔH~2.7 J/g) was visible in the DSC trace after its processing, indicating crystallisation.

Also, in the case of P(iPrOx–nPrOx)_12k_ after extrusion from melt, an endothermic peak could be seen, indicating crystallisation of the polymer during processing. However, in this case, crystallisation was related to prolonged heating of POx at elevated temperature.

As can be seen, the processing of (co)poly(2-oxazoline)s by various techniques significantly influenced both their stability and thermal and crystalline properties. Among examined POx, P(iPrOx-nPrOx) seems more attractive than PiPrOx for the formation of matrices that could have potential application as scaffolds in biomedical engineering. This is due to ease of processing of the copolymer and stability upon processing conditions (unless it is annealed for prolonged time). Moreover, obtained copolymer-based materials are more elastic and contain less amount of crystalline fraction, what is important having in a mind their potential application. The utility of the obtained materials as scaffolds is in progress.

## 4. Conclusions

Materials in the form of non-woven fibrous mats and three-dimensional moulds of strictly defined properties based on PiPrOx and gradient copolymers of iPrOx with nPrOx were obtained by electrospinning and FDM techniques.

In the case of electrospinning, for the first time, poly(2-oxazoline)s that were different than PEtOx were utilised for the fabrication of non-woven mats. Electrospinning conditions were optimised for POx of relatively low molar masses (from 12,600 to 51,000 g/mol) and low dispersity (*Ð* less than 1.32) for both water and hexafluoro-2-propanol solutions. It was established that homogeneous, non-beaded fibre mats could be obtained when PiPrOx with a molar mass of 21,000 g/mol was electrospun at a concentration above 60 wt %. However, this relatively high concentration in water could be decreased when PiPrOx of a higher molar mass was applied (50 wt % at 42,000 g/mol) or when the solvent was changed from aqueous to an organic solvent (HFP) (15 wt % at 21,000 g/mol and 10 wt % at 42,000 g/mol). PiPrOx non-woven mats were stiff and fragile, and the relative value of Young’s modulus was more than three times higher than for the P(iPrOx–nPrOx) mats. 

With FDM, PiPrOx and P(iPrOx–nPrOx) were processed to form materials of strictly defined size, shape, and porosity. Multi-layer moulds of cylindrical and cubical shape were obtained. Copolymerisation of 2-isopropyl-2-oxazoline with 2-n-propyl-2-oxazoline allowed for the processing temperature to be lowered to 160 °C. Due to applying a higher temperature for PiPrOx, the polymer degraded during processing and blocked the extrusion nozzle. The copolymer P(iPrOx–nPrOx) has already a suitable extrusion viscosity at 160 °C, which, together with the significantly reduced crystallisation ability, allowed us to obtain mouldings in an effective way.

To determine the processability and stability of the obtained materials, the molar masses and thermal properties of POxs after processing were determined. The molar mass of all (co)poly(2-oxazoline)s did not change after electrospinning. Also, FDM did not influence the molar masses of the (co)polymers; however, long processing of the material caused degradation and an increase in molar mass dispersity to 1.7.

The thermal properties of all (co)poly(2-oxazoline)s changed significantly after electrospinning. The content of the crystalline phase increased what was observed as enlargement of both exo- and endothermic peaks in DSC traces for PiPrOx, and appearance of endothermic peak in case of P(iPrOx–nPrOx). This observation is rather surprising and requires more studies. The presence of melting peaks in the DSC of (co)poly(2-oxazoline)s after extrusion from melt can be explained by the crystallisation of the polymer at its processing temperature.

We hope that this comparison of the properties of the starting polymer and the final material after processing will be helpful when designing new materials with strictly defined parameters.

## Figures and Tables

**Table 1:** Characterisation data of the obtained iPrOx-based (co)polymers.

Symbol	iPrOx:nPrOx ^a^	*M*_theoret_	*M*_n_^b^ (g/mol)	*Ð*	DP ^c^	*T*_CP_^d^ (°C)	*T*_g_^e^ (°C)	*T*_m_^e^ (°C)
PiPrOx_21k_	100:0	20 000	21 400	1.01	180	37	70	205
PiPrOx_42k_	100:0	40 000	42 000	1.06	370	35	70	205
P(iPrOx-nPrOx)_12k_	48:52	10 000	12 600	1.06	110	29	50	–
P(iPrOx-nPrOx)_51k_	47:53	52 000	51 000	1.32	450	27	50	–

^a^ Based on ^1^H NMR, ^b^ Obtained by GPC MALLS, ^c^ Based on monomer feed and ^1^H NMR, ^d^ Obtained by turbidimetric measurements (UV–Vis), ^e^ Obtained by DSC.

## Supplementary Materials

The following are available online at https://www.mdpi.com/2073-4360/12/2/295/s1, Figure S1: Electrospinning system used for the preparation of PiPrOx and P(iPrOx-nPrOx) non-woven mats: (a) utilised set-up, (b) schematic representation of the electrospinning system. Figure S2. The Bioscaffolder system used for extrusion from melt. Figure S3. ^1^H NMR spectra of (a) PiPrOx_42k_, (b) P(iPrOx–nPrOx)_12k_ and (c) P(iPrOx–nPrOx)_51k_ (CDCl_3_, 600MHz). Figure S4. DSC trace of PiPrOx_21k_, heating rate of 10°C/min. Figure S5. AFM micrographs of (a) PiPrOx_42k_ and (b) P(iPrOx–nPrOx)_51k_ fibres used for the analysis of relative values of Young’s modulus.
