# Supplementary material for: Processing of (Co)Poly(2-oxazoline)s by Electrospinning and Extrusion from Melt and the Postprocessing Properties of the (Co)Polymers
Source: Polymers (Basel). 2020 Feb 2;12(2):295. doi: 10.3390/polym12020295 (PMC7077476; doi:10.3390/polym12020295)
Supplement: Supplementary file 1 [file polymers-12-00295-s001.pdf]

# Supplementary Materials: Processing of (co)poly(2-oxazoline)s by electrospinning and extrusion from melt and the post-processing properties of the (co)polymers

**Wojciech Wałach [1,*], Natalia Oleszko-Torbus [1], Alicja Utrata-Wesołek [1], Marcelina Bochenek [1], Ewa Kijeńska-Gawrońska [2], Żaneta Górecka [2], Wojciech Święszkowski [2], Andrzej Dworak [1]**

[1] Centre of Polymer and Carbon Materials, Polish Academy of Sciences, 34 M. Curie-Skłodowskiej St., 41-819 Zabrze, Poland; wwalach@cmpw-pan.edu.pl (W.W); noleszko@cmpw-pan.edu.pl (N.O.T.); autrata@cmpw-pan.edu.pl (A.U.W.); mbochenek@cmpw-pan.edu.pl (M.B); adworak@cmpw-pan.edu.pl (A.D)
[2] Faculty of Materials Science and Engineering, Warsaw University of Technology, 141 Woloska St., 02-507 Warsaw, Poland; ewa.kijenska@pw.edu.pl (E.K.G.); gorecka.zaneta@gmail.com (Ż.G.); wojciech.swieszkowski@inmat.pw.edu.pl (W.Ś.)
* Correspondence: wwalach@cmpw-pan.edu.pl (W.W); Tel.: +48 32 271 60 77 (ext. 259)

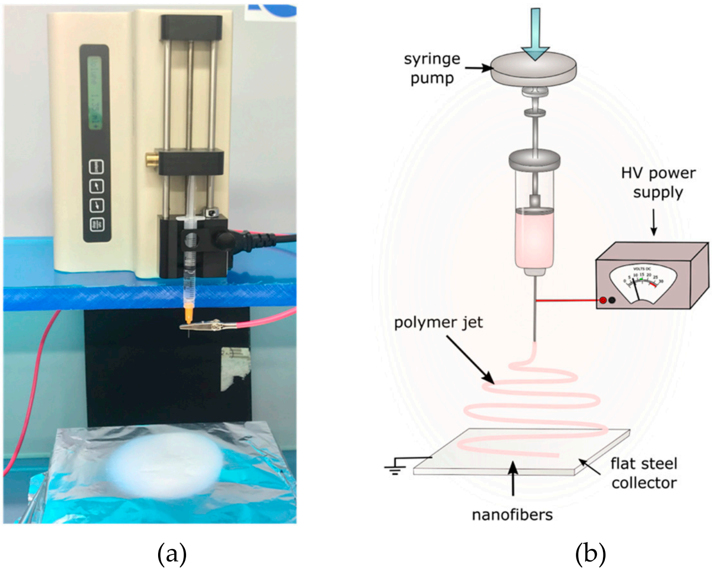


**Figure S1.** Electrospinning system used for the preparation of PiPrOx and P(iPrOx-nPrOx) nonwovens: (a) utilized set-up, (b) schematic representation of the electrospinning system.

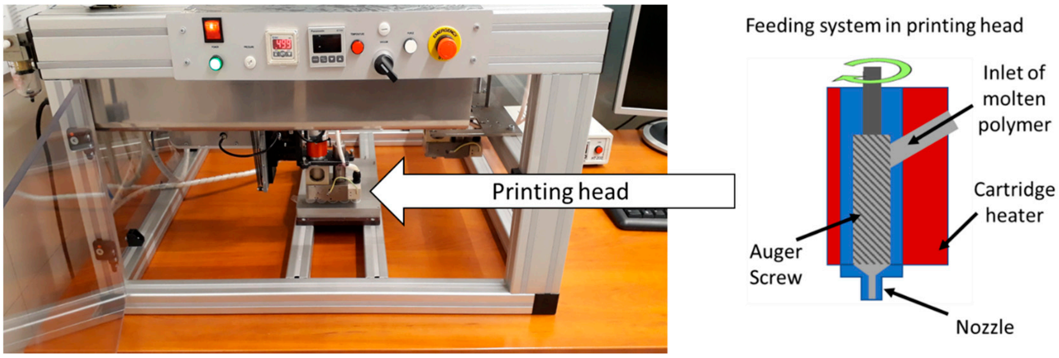


**Figure S2.** The Bioscaffolder system used for extrusion from melt.

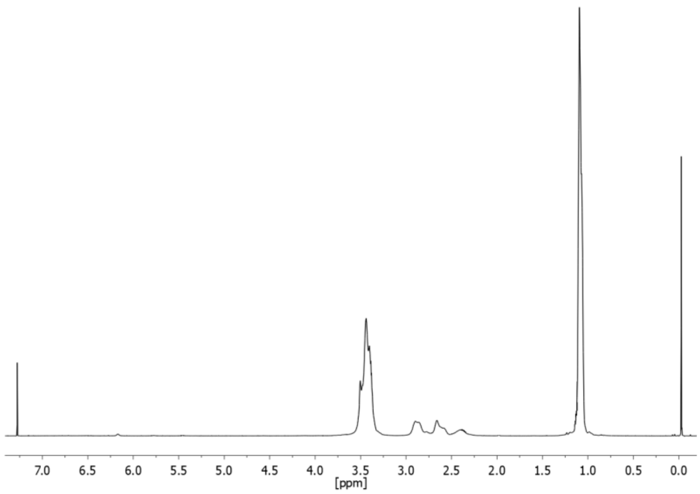


(a)

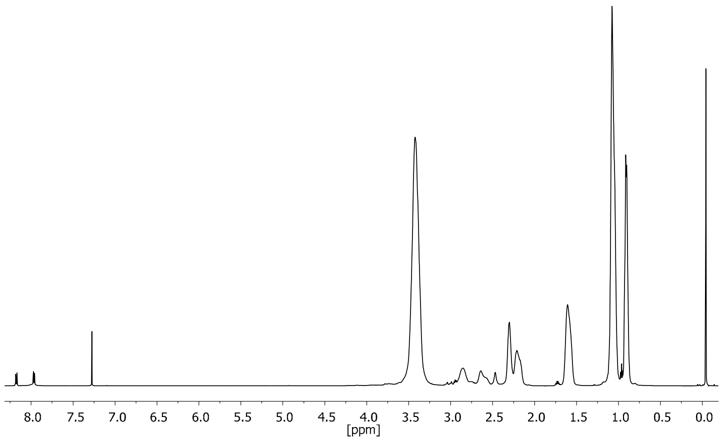


(b)

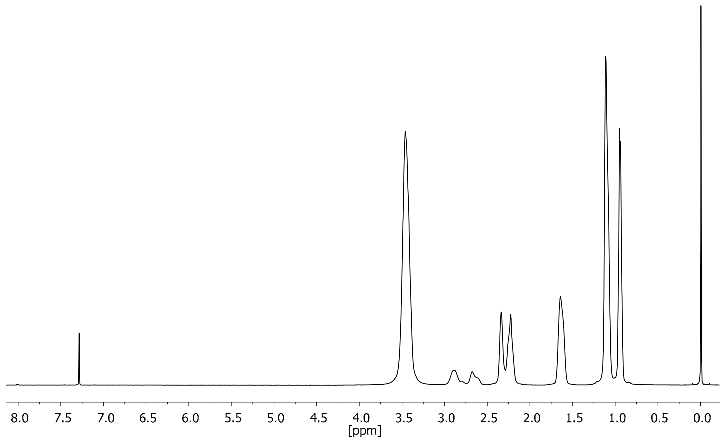


(c)

**Figure S3.** $^1$H NMR spectra of (a) $PiPrOx_{42k}$, (b) $P(iPrOx–nPrOx)_{12k}$ and (c) $P(iPrOx–nPrOx)_{51k}$ ($CDCl_3$, 600MHz).

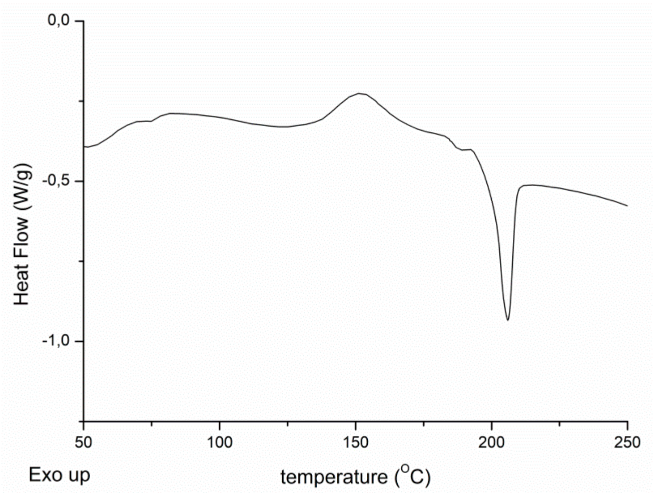


**Figure S4.** DSC trace of $PiPrOx_{21k}$, heating rate of 10°C/min.

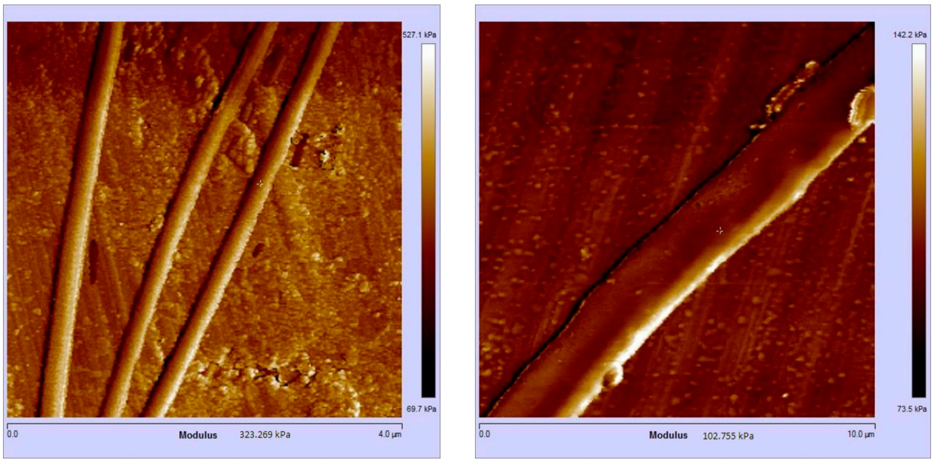


(a) (b)

**Figure S5.** AFM micrographs of (a) $PiPrOx_{42k}$ and (b) $P(iPrOx–nPrOx)_{51k}$ fibers used for the analysis of relative values of Young's modulus.
